# A novel hollow iron nanoparticle system loading PEG-Fe_3_O_4_ with C5a receptor antagonist for breast cancer treatment

**DOI:** 10.3389/fimmu.2024.1466180

**Published:** 2024-10-17

**Authors:** Hong Yang, Guiqing Li, Ji Zhang, Jing Zhao, Yunpei Zhao, Yufei Wu, Zihan Sun, Shuangshuang Song, Ying Zou, Zhihao Zou, Xiao Han, Boshao Deng, Lulu Wang, Hang Rao, Guilian Xu, Shufeng Wang, Sheng Guo, Huanyu Ding, Yan Shi, Yuzhang Wu, Jian Chen

**Affiliations:** ^1^ Department of Immunology, Army Medical University (Third Military Medical University), Chongqing, China; ^2^ Biomedical Analysis Center, Army Medical University (Third Military Medical University), Chongqing, China; ^3^ Department of Cardio-renal, Chinese People’s Liberation Army 74th Group Military Hospital, Guangzhou, China; ^4^ Breast Disease Center, Third Affiliated Hospital of Chongqing Medical University, Chongqing, China; ^5^ The First Affiliated Hospital of Army Military Medical University, Department of General Practice, Chongqing, China; ^6^ Department of General Surgery, First Affiliated Hospital, Army Medical University (Third Military Medical University), Chongqing, China; ^7^ Institute of Medical Technology, Chongqing Medical and Pharmaceutical College, Chongqing, China

**Keywords:** C5a/C5aR pathway, ferroptosis resistance, iron nanoparticles, PEG- Fe_3_O_4_@C5aRA, macrophage polarization

## Abstract

Breast cancer is the most diagnosed malignancy and major cause of cancer death among women population in the worldwide. Ferroptosis is a recently discovered iron-dependent regulated cell death involved in tumor progression and therapeutic response. Moreover, increasing studies have implied that ferroptosis is a promising approach to eliminating cancer cells like developing iron nanoparticles as a therapeutic agent. However, resistance to ferroptosis is a vital distinctive hallmark of cancer. Therefore, further investigation of the mechanism of ferroptosis resistance to enhance its tumor sensitivity is essential for ferroptosis-target breast cancer therapy. Our results revealed that the activation of C5a/C5aR pathway can drive resistance to ferroptosis and reshaping breast cancer immune microenvironment. Accordingly, loading PEG-Fe_3_O_4_ with C5aRA significantly improved the anti-tumor effect of PEG- Fe_3_O_4_ by inhibiting ferroptosis resistance and increasing macrophage polarization toward M1 phenotype. Our findings presented a novel cancer therapy strategy that combined cancer cell metal metabolism regulation and immunotherapy. The study also provided support for further evaluation of PEG- Fe_3_O_4_@C5aRA as a novel therapeutic strategy for breast cancer in clinical trials.

## 1 Introduction

Breast cancer is the most diagnosed cancer among women population in the worldwide. In 2020, an estimated 2.3 million new cases and >685,000 deaths caused by breast cancer are reported (1). Although survival rates have markedly improved over the past two decades, the incidence of this disease continues to rise worldwide (2). So, it makes sense to develop new initiatives and strategies focused on the prevention and treatment of breast cancer.

Ferroptosis, a kind of programmed cell death, is a hot topic in the field of cancer research. It is characterized by the iron-mediated generation of reactive oxygen species (ROS), also known as the Fenton reaction. ROS interacts with polyunsaturated fatty acids (PUFAs) on membranes, leading to lipid peroxidation and, ultimately, membrane rupture (3). For the increased metabolism and cell proliferation, cancer cells were highly reliant on iron content and experience a state of hyper oxidation. Ferroptosis-based cancer therapy is expected to overcome the limitations of traditional treatments mediated by apoptosis pathway (4).

Recently, iron nanoparticles such as ferumoxytol, a Food and Drug Administration (FDA)-approved iron supplement drug, has been proved to have therapeutic effect on the growth of early breast cancers, lung cancer metastases and prostate cancer (5). And one kind of iron nanoparticles, mesoporous hollow iron nanoparticles have attracted great attention in tumor therapy field for its good ability as a drug delivery carrier (6, 7). Iron nanoparticles could serve as an iron source, massively increasing the cellular levels of irons, lead cancer cells to ferroptosis. In addition to targeting cancer cells, iron nanoparticles are also relevant to the polarization of tumor-associated macrophages, which have been explored as potential targets in cancer immunotherapy. Increasing evidence suggests that alterations in the iron metabolic profile of macrophages influence their activation state and biological functions (8). The unique properties of inducing ferroptosis in cancer cells and altering macrophage polarization have positioned them as potential candidates in the field of cancer therapy. Furthermore, numerous studies have specifically investigated the use of iron nanoparticles to inhibit tumor progression in breast cancer and improve the effectiveness of cancer treatment strategies (9–11).

However, the iron nanoparticles alone are insufficient to induce lethal ferroptosis due to the presence of ferroptosis resistance in cancer cells and concerns of safety. So, the investigation of new ferroptosis-based therapeutic with high efficiency and low systemic toxicity for cancer therapy is needed. Besides, although iron nanoparticles showed great promise in cancer therapy, many promising candidates previously approved by the Food and Drug Administration have been withdrawn due to hypersensitivity and toxicity concerns. One of the major drawbacks of iron nanoparticles is the lack of biocompatibility with blood components and the immune system (12, 13). It has been reported PEG-Fe_3_O_4_ iron oxide nanoparticles could activate the complement system and induce an inflammatory response (14).

Complement elements such as C3a or C5a appears to participate in some processes of the tumor progression, including the regulation of tumor angiogenesis and immune cells recruitment and phenotype (15, 16). Indeed, C5a was demonstrated to promote tumor metastasis of breast cancer by altering T-cell responses in the metastatic niche (17). Besides, our previous research also provided evidence that breast cancer development may rely on C5a/C5aR interaction, for which MAPK/p38 pathway participated in downregulating the p21 expression. And C5a receptor antagonists (C5aRA) exhibited significant attenuation of BC cell growth in mouse model (18). And it was demonstrated C5a/C5aR pathway could upregulated NRF2 in myeloma cells, which suggested it may be related to breast cancer cell ferroptosis resistance (19).

**Figure 1:**
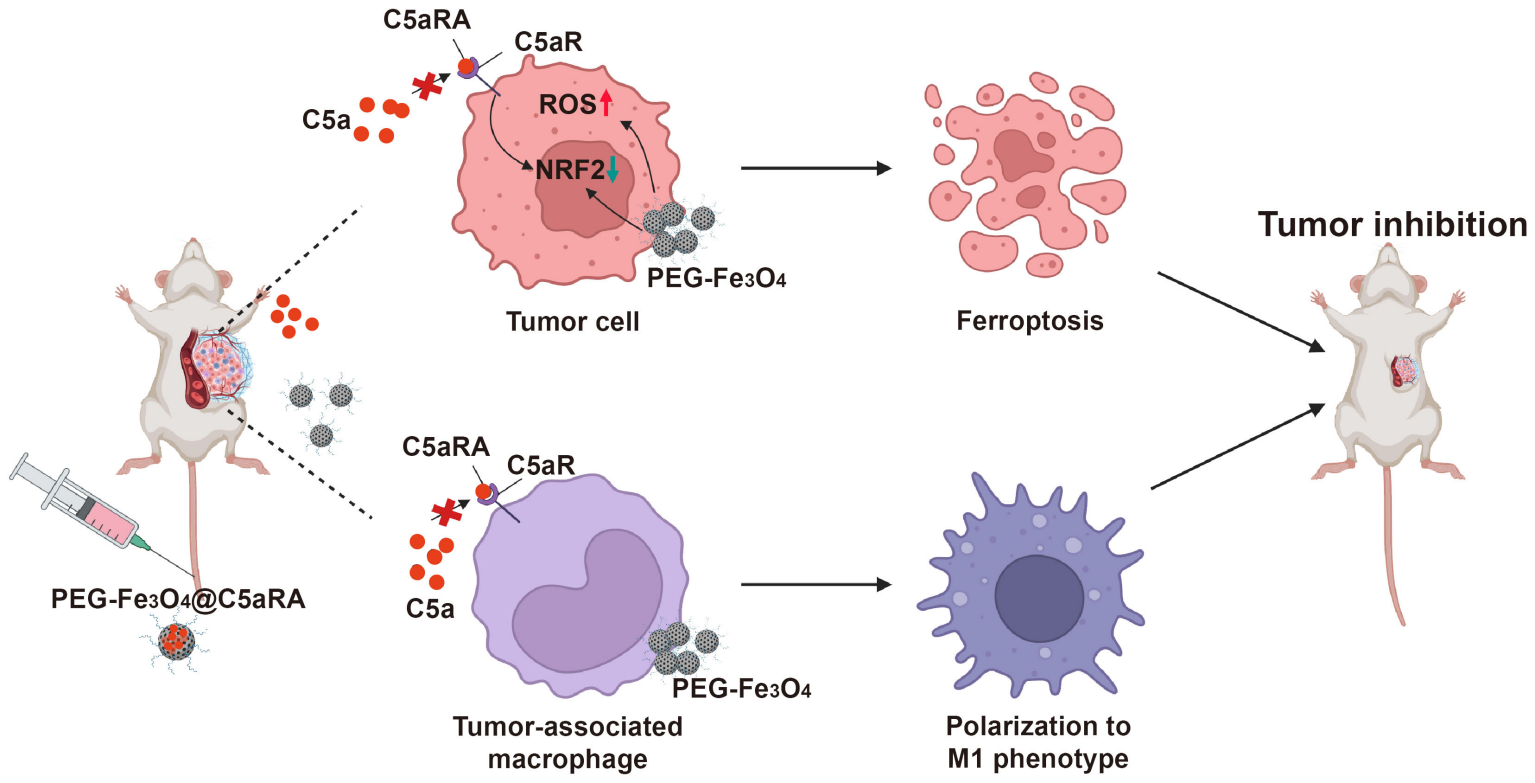
Schematic illustration of superior anti-tumor effect of PEG-Fe_3_O_4_@C5aRA through ferroptosis and macrophages polarization to M1 phenotype.

Based on these, we designed a C5aRA-loading porous PEG-Fe_3_O_4_, which was termed PEG-Fe_3_O_4_@C5aRA. This system enables the augmentation of PEG-Fe_3_O_4_ induced cancer cell ferroptosis and tumor immunotherapy. As shown in **Figure 1**, our research demonstrated that the release of C5aRA by PEG-Fe_3_O_4_@C5aRA promoted PEG-Fe_3_O_4_ cancer therapy ability not only by promoting macrophage polarization to M1 phenotype but also reducing ferroptosis resistance through down-regulating ferroptosis key regulator protein NRF2. This study provided a novel insight into the development of a novel nano-system that synergized various therapeutic mechanisms for breast cancer treatment. The complete workflow of this study is depicted in **Figure 1**.

## 2 Materials and methods

### 2.1 Patients and clinical specimens

BC tissues and tumor-adjacent tissues (5 cm from the tumor margin) were obtained from 44 patients who underwent BC surgery, between 2008 and 2010, with lymph node dissection at the Chongqing Southwest Hospital (China). None of the patients underwent preoperative chemotherapy or radiation therapy. 9 from stage I breast cancer patients, 14 from stage II breast cancer patients, and 21 from stage III breast cancer patients. All study procedures were conducted with the pre-approval of the Ethics Committee of the Third Military Medical University. All the patients were informed about the sample processing steps and provided their written consent prior to the study enrollment.

### 2.2 Construction of PEG-Fe_3_O_4_@C5aRA

1mg hollow mesoporous PEG-Fe_3_O_4_ (Nan Jing Jike Biotechnology, China) was mixed with 1mg C5aRA (GL Biochem, China), and the final volume of the mixture was 2mL. This mixture was kept under stirring at 4°C for 48 h. The loaded nanoparticles, PEG-Fe_3_O_4_@C5aRA, were decanted by centrifugation at 12,000× g for 30min.

### 2.3 Animals study

All animal experiments were performed in compliance with institutional guidelines for use and care of animals. Wild-type (WT) BALB/C mice were obtained from the Animal Institute of Academy of Medical Science (Beijing, China). C5aR-KO mice (BALB/C background) were purchased from Jackson Laboratory (Ellsworth, Maine, US). BALB/C mice were 6-8 weeks old at the beginning of the experiments, and the groups were matched by age and sex. All mice were housed in the individual ventilated cages at the Institute of Immunology of the Army Medical University (Chongqing, China).

To evaluate the role C5a/C5aR pathway in breast cancer progression, two experimentation groups are set: 1) 4T-1 xenograft female BALB/C mice; 2) 4T-1 xenograft female C5aR-KO mice.

To figure out the effect of PEG-Fe_3_O_4_ on tumor progression, 4T-1 xenograft female BALB/C mice were randomly assigned into two experimentation groups: 1) treated with sterile 0.9% NaCl solution; 2) exposed to PEG-Fe_3_O_4_ (20 mg/kg). Mice were intravenously injected with sterile 0.9% NaCl solution or PEG-Fe_3_O_4_ by the lateral tail vein on day5, day7 and day9.

To test whether PEG-Fe_3_O_4_ could activate complement system, female BALB/C mice were randomly assigned into two experimentation groups (ten mice per group): 1) control (sterile 0.9% NaCl solution); 2) exposed to PEG-Fe_3_O_4_ (20 mg/kg). The selected dose of 20 mg/kg of body weight agreed with the dose applied in therapeutic studies of PEG-Fe_3_O_4_. We chose 90 minutes as exposure time to evidence an increment of the complement markers. Plasma concentrations of C5a was determined by ELISA with commercial kits.

Tumor model: For 4T-1 breast cancer models, 4T-1 cells (1 × 10^6^) were suspended in 0.1 ml of phosphate-buffered saline (PBS) media and injected subcutaneously into the right back of 6-8 weeks female mice on day 0.

To evaluate the potential of PEG-Fe_3_O_4_@C5aRA on inhibition of breast tumor growth, 4T-1 xenograft female BALB/C mice were randomly assigned into four experimentation groups: 1) control (sterile 0.9% NaCl solution); 2) exposed to PEG-Fe_3_O_4_ (20mg/kg); 3) exposed to C5aRA (1mg/kg); 4) exposed to PEG-Fe_3_O_4_@C5aRA (20 mg/kg). Tumor volumes were measured in two dimensions using a caliper and calculated by the formula (length × width^2^/2) (20)

For *in vivo* bioluminescence analysis: 4T-1-LUC cells (1 × 10^6^) were suspended in 0.1 ml of 1 × PBS media and injected into the right back of BALB/C female mice on day 0. The mice were randomly assigned into four experimentation groups: 1) control (sterile 0.9% NaCl solution); 2) exposed to PEG-Fe_3_O_4_ (20mg/kg); 3) exposed to C5aRA (1mg/kg); 4) exposed to PEG-Fe_3_O_4_@C5aRA (20 mg/kg). The tumor growth was regularly assessed imaging at day11, 13, 15. Luciferase activity was measured by the IVIS Spectrum *in vivo* imaging system (PerkinElmer) according to the manufacturer’s instructions.

### 2.4 MR imaging

The tumor-bearing mice were intravenously injected with PEG-Fe_3_O_4_@C5aRA (20 mg/kg), after injection for 4 hours, MR imaging was conducted on a 1.0-T clinical MRI scanner (Aspect M7, Israel).

### 2.5 Biological safety assay

The tumor-bearing mice were intravenously injected with PEG-Fe_3_O_4_@C5aRA (20 mg/kg), after injection for 48 hours, mice serum was obtained to test the main liver function index GOT (Sangon Biotech, Cat#D799581-0050) and GPT (Sangon Biotech, Cat#D799579-0050) according to the manufacturer’s instructions.

### 2.6 Histopathological analysis

After different treatments, mice from different groups were euthanized by CO_2_ inhalation. The main organs of the mice, such as their hearts, livers, spleens, kidneys, lungs and tumors, were collected and fixed in 10% neutral buffered formalin for 48 h. Then, these tissues were embedded in paraffin after being processed by alcohol and xylene and sectioned into 5 μm thickness.

### 2.7 Immunohistochemistry assay

Tumor Tissue sections were incubated with a primary antibody against NRF2 (Proteintech, Cat#16396-1-AP, Rabbit), Glutathione peroxidase 4 (GPX4) (Proteintech, Cat#67763-1-Ig, mouse), C5aR (Proteintech, Cat#21316-1-AP, Rabbit), C5b-9 (Abcam, Cat#ab55811, Rabbit), C5 (Beyotime, Cat#AF6360, Rabbit). HRP-labeled Goat Anti-Rabbit IgG(H+L) (Beyotime, Cat#A0208) and HRP-labeled Goat Anti-Mouse IgG(H+L) (Beyotime, Cat#A0216) as secondary antibody. The peroxidase activity was visualized with diaminobenzidine tetrahydroxy chloride (DAB) solution. The sections were counter stained with hematoxylin. Dark brown staining was considered positive.

### 2.8 Hematoxylin and eosin stain

Organ tissues were stained with haematoxylin and eosin (C0105, Beyotime) according to the manufacturer’s instructions.

### 2.9 Perl’ staining

Tumor tissue sections were stained for iron using Prussian Blue Iron Stain Kit (Solarbio, Cat#G1424) following manufacturer’s instructions. Perls’ blue staining was further enhanced using the DAB peroxidase substrate kit SK-4100 (Vector Labs).

### 2.10 Single cell suspension of tissue and flow cytometry analysis

Tumor tissues were digested with Tumor Dissociation Kit (Miltenyi Biotec, Cat#130-096-730) using gentleMACS™ Octo Dissociator with Heaters (Miltenyi Biotec) and then crushed through mesh for single cell suspension. To determine macrophage polarization, cells from tumor tissues were stained with Zombie.NIR™ Dye (BioLegend, Cat#B323327), anti-mouse CD45 (BioLegend, Cat#103132), CD11b (BioLegend, Cat#101263), F4/80 (BioLegend, Cat#123110), I-A/I-E (BioLegend, Cat#107605), and CD206 (BioLegend, Cat#141717). After staining process by following the manufacturer’s instructions, samples were analyzed by flow cytometry (CytoFLEXTM).

### 2.11 Cell lines and culture conditions

The human MCF-7 and MCF10A lines were obtained from the Cell Bank of the Chinese Academy of Sciences (Shanghai, China). The mouse 4T-1 and HC11 cell lines were obtained from Procell Life Science and Technology. MCF-7, MCF-10A and 4T-1 were routinely cultured in Dulbecco’s Modified Eagle’s Medium (high glucose) (Gibco, Life Technologies, USA) supplemented with 10% fetal bovine serum (Gibco, Life Technologies, USA) and maintained at 37°C in a humidified incubator with 5% CO_2_. HC11 was cultured in RPMI-1640 Medium (Gibco, Life Technologies, USA) supplemented with 10% fetal bovine serum (Gibco, Life Technologies, USA) and maintained at 37°C in a humidified incubator with 5% CO_2_.

### 2.12 Cell treatment

To investigate the role of C5a/C5aR pathway on NRF2 expression, 4T-1 was stimulated with 480 ng/mL C5a (Biovision, USA) for 30 min. For the other group, before stimulation with C5a, these cells were pre-treated with 10 nM C5aRA for 1 hour. The expression of NRF2 was assayed by western blot.

To investigate the role of C5a/C5aR pathway on ferroptosis resistance, four experiments groups are set. 1) control group; 2) the 4T-1 cells were treated with 2ug/mL Sorafenib (Targetmol, Cat# 284461-73-0) for 48 hours; 3) the 4T-1 cells were stimulated with 2ug/mL Sorafenib and 480 ng/mL C5a; 4) before stimulation with 480 ng/mL C5a and 2ug/mL Sorafenib, these cells were pre-treated with 10 nM C5aRA for 1 hour. Cell viability and intracellular ROS was measured. The expression of GPX4 and NRF2 were evaluated by western blot.

To determine the role of C5a/C5aR pathway on macrophage polarization, RAW264.7 or PMA (100ng/mL PMA, 48h) (Sigma, Cat#P1585-1MG) pre-treated THP-1 was stimulated with 480 ng/mL C5a (Biovision, USA) for 30 min. For the other group, before stimulation with C5a, these cells were pre-treated with 10 nM C5aRA for 1 hour.

To evaluate the influence of Fe_3_O_4_ on cell, 200ug/mL or 400ug/mL Fe_3_O_4_ was treated on cells for 48h, and then cell viability was assayed. The expression of GPX4 and NRF2 were evaluated by western blot.

To evaluate the potential role of PEG-Fe_3_O_4_@C5aRA on 4T-1, four experiments groups are set. 1) control group; 2) the 4T-1 cells were treated with 200ug/mL PEG-Fe_3_O_4_ for 48 hours; 3) the 4T-1 cells were stimulated with 480 ng/mL C5a (Biovision, USA) and 200ug/mL PEG-Fe_3_O_4_ for 48 hours; 4) the 4T-1 cells were stimulated with 200ug/mL PEG-Fe_3_O_4_@C5aRA, and after 1 hour, 480 ng/mL C5a was added. The intracellular ROS was measured and the expression of GPX4 and NRF2 was evaluated by western blot.

To investigate the role of PEG-Fe_3_O_4_ on macrophage polarization, RAW264.7 or PMA (100ng/mL PMA, 48h) pre-treated THP-1 was stimulated with 200ug/mL PEG-Fe_3_O_4_ for 48 hours.

To evaluate the potential role of PEG-Fe_3_O_4_@C5aRA on macrophage polarization, four experiments groups are set. 1) control group; 2) the cells were treated with 200ug/mL PEG-Fe_3_O_4_ for 48 hours; 3) the cells were stimulated with 480 ng/mL C5a and 200ug/mL PEG-Fe_3_O_4_ for 48 hours; 4) the 4T-1 cells were stimulated with 200ug/mL PEG-Fe_3_O_4_@C5aRA for 1 hour and 480 ng/mL C5a was added for 48 hours.

### 2.13 Cell viability assay

After different treatments, cells were replaced with fresh medium containing 1 mg/mL MTT (MCE, Cat#HY-Y0320) and then incubated at 37°C for 2 h. Crystals were dissolved in dimethyl sulfoxide, and the optical absorbance at 570 nm was measured.

### 2.14 Intracellular ROS measurement

To measure intracellular ROS level, cells (5 × 10^5^ per well) were seeded in 12-well plates. After different treatments, cells were detached with trypsin, washed twice with PBS, and then incubated with 10 µM DCFH-DA (Beyotime, Cat#S0033S) at 37°C for 20 min in the dark, mix upside down every 3-5 minutes. After washing with PBS for three times. Change of fluorescence intensity in the solution was measured upon excitation wavelength at 488 nm and the emission spectra was recorded from 500 to 600 nm. The fluorescence intensity at 525 nm (λmax) was plotted against the irradiation time. Rosup (500 μg/mL) acts as a positive control for ROS production.

### 2.15 Protein extraction and western blotting

Cells or tumor tissues were lysed in T-PER™ Tissue Protein Extraction Reagent (Thermo, Cat#78510) containing protease inhibitors cocktail (CWBIO, Cat#CW2200S). Lysates were centrifuged at 12,000 rpm for 15 min. Protein extracts were solubilized in loading buffer (Bioground, Cat#BG0022). Equal amounts of lysate were separated on SDS-PAGE (ExpressPLUS™, Cat#M42015C) and transferred onto a 0.45μm polyvinyl difluoride (PVDF) membrane (Beyotime, Cat#FFP39). The protein was identified by incubating the membrane with primary antibodies followed by horseradish peroxidase-conjugated secondary antibodies. The primary antibodies contain NRF2 (Proteintech, Cat#16396-1-AP, Rabbit, 1:1000), GPX4 (Proteintech, Cat#67763-1-Ig, mouse, 1:1000), β-actin (Beyotime, Cat#AF5003, Rabbit). HRP-labeled Goat Anti-Rabbit IgG(H+L) (Beyotime, Cat#A0208) and HRP-labeled Goat Anti-Mouse IgG(H+L) (Beyotime, Cat#A0216) as secondary antibodies.

### 2.16 RNA extraction and RT-qPCR assays

After treatment, total RNA was extracted using RNA Extraction Kit (Beyotime, Cat#R0026). Purified RNA was converted into cDNA by reverse transcription. RT-qPCR was performed with SYBR Green Master Mix (Invitrogen) using the StepOnePlus™ Real-Time PCR system (Life Technologies, Carlsbad, CA, USA). Expression levels were normalized with internal control β-actin. The primer sequences of mouse *iNOS*: Forward 5’-3’CTGCAGCACTTGGATCAGGAACCTG,Reverse3’-5’GGGAGTAGCCTGTGTGCACCTGGAA; mouse *Arg1*: Forward 5’-3’CAGAAGAATGGAAGAGTCAG, Reverse3’-5’CAGATATGCAGGGAGTCACC; mouse mouse *CCL3*: Forward 5’-3’CATATGGAGCTGACACCCCG, Reverse3’-5’GAGCAAAGGCTGCTGGTTTC; human *CCR7*: Forward 5’-3’TGGTGGTGGCTCTCCTTGTC, Reverse3’-5’TGTGGTGTTGTCTCCGATGTAATC; human *CCL17*: Forward 5’-3’CGGGACTACCTGGGACCTC, Reverse3’-5’CCTCACTGTGGCTCTTCTTCG.

### 2.17 Statistical analysis

Statistical analysis was conducted using GraphPad Prism (version 8.0) software. The statistical significance for comparisons of two groups was evaluated using the student’s t-test. Statistical significance for comparisons of three or four groups was determined by one-way ANOVA. All data were presented as the mean ± standard error of mean (SEM). p < 0.05 was considered statistically significant. Statistical significance was denoted (no significance [NS], *p < 0.05; **p <0.01; ***p < 0.001, ****p<0.0001.) in the figures and figure legends. All experiments were independently repeated at least thrice.

## 3 Results

### 3.1 C5a/C5aR pathway was activated in breast cancer, and it could induce ferroptosis resistance

**Figure 2:**
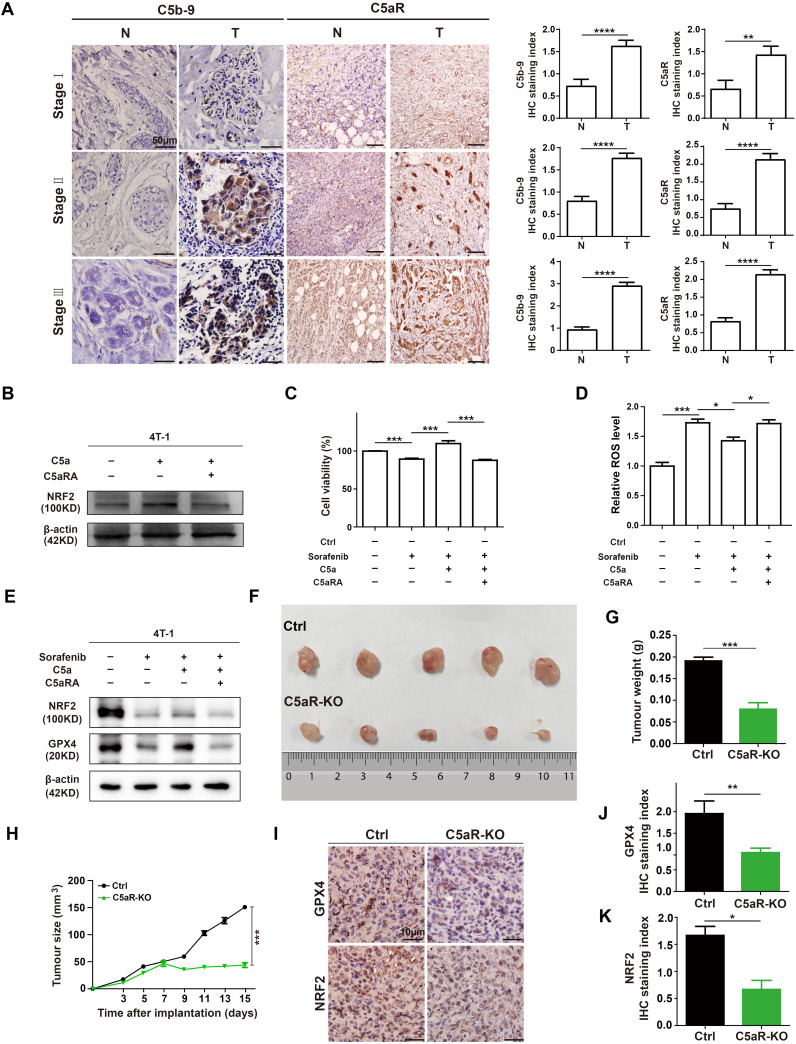
C5a-C5aR pathway activation in human breast cancer and its role in ferroptosis resistance and breast cancer progression in 4T-1 xenograft mouse model. **(A)**, the expression of C5b-9 and C5aR in BC tumoral tissues (T) and BC-adjacent non-tumoral tissues (N) of patients with different clinical stages were evaluated by immunohistochemistry. Statistical analyses of the difference of C5b-9 and C5aR expression between different groups were presented in the right. **(B)** Immunoblotting of NRF2 in 4T-1 cells after treatment with 480 ng/mL C5a or C5a plus 10 nM C5aRA for 48h. **(C-E)**, Relative cell viability **(C)**, ROS level **(D)**, NRF2 and GPX4 expression analysis **(E)** of 4T-1 cells after with different treatment. 1) control group; 2) the 4T-1 cells were treated with 2ug/mL Sorafenib for 48 hours; 3) the 4T-1 cells were stimulated with 2ug/mL Sorafenib and 480 ng/mL C5a; 4) before stimulation with 480 ng/mL C5a and 2ug/mL Sorafenib, these cells were pre-treated with 10 nM C5aRA for 1 hour. Mice were implanted with 1.0 × 10^6^ 4T-1 cells in the mammary fat pad. The tumor growth was measured by representative tumor images **(F)**, tumor weight **(G)**, and tumor size **(H)** (n=5). **(I-K)**, Immunohistochemistry and statistical analyses of the expression of GPX4 and NRF2 in tumor tissues. Statistical differences are assessed by student’s t-test. Data were mean ± s.e.m. *, p < 0.05, **, p < 0.01, ***, p < 0.001, ****, p < 0.001.

To explore the role of complement in breast cancer, the C5b-9 and C5aR expression were analyzed in the tumoral tissues from BC patients at different clinical stages by IHC. C5b-9 and C5aR expression were significantly higher in the tumoral tissues than in the BC-adjacent non-tumoral tissues (**Figure 2A**). As previous study has proved that C5a/C5aR pathway could up-regulate the key ferroptosis related gene NRF2 (19), we speculated that it may regulate iron metabolism. For investigating the relationship between C5a/C5aR pathway and iron metabolism, 4T-1 cells were treatment with C5a. NRF2 was observed up-regulated expression after C5a administration, and the up-regulation was reversed after treatment with C5aRA (**Figure 2B**). Since C5a/C5aR pathway induced upregulated NRF2 expression, and previous studies have proved that NRF2 is a key regulator of the cellular antioxidant response and ferroptosis (21), we then investigated whether C5a/C5aR pathway could even partially induce ferroptosis resistance. Hence, ferroptosis on 4T-1 cells was induced by Sorafenib, a ferroptosis inducer (22). As presented in **Figure 2C**, 4T-1 cell death induced by Sorafenib could be reversed by C5a. Besides, ferroptosis sigh ROS was reduced after C5a treatment (**Figure 2D**). The western blot results showed C5a elevated the expression of NRF2 and GPX4 that were downregulated by Sorafenib administration (**Figure 2E**). These *in vitro* results indicated ferroptosis resistance induced by C5a/C5aR pathway, which could partially explain how breast cancer cells overcome ferroptosis.

In our *in vivo* study, female BALB/C and C5aR-KO mice were transplanted with 4T-1 cells to further evaluate the role of C5a/C5aR pathway on breast cancer progression. C5aR deficiency caused a significant reduction in the tumor growth (**Figures 2F-H**). The IHC staining of xenografts tumor tissues showed that the protein levels of NRF2 and GPX4 were significantly reduced in C5aR-KO mice (**Figures 2I-K**). In conclusion, C5a/C5aR pathway could promoted breast cancer progression and lead ferroptosis resistance *in vitro* and *in vivo*.

### 3.2 C5a/C5aR pathway promoted macrophage polarization to M2 phenotype

**Figure 3:**
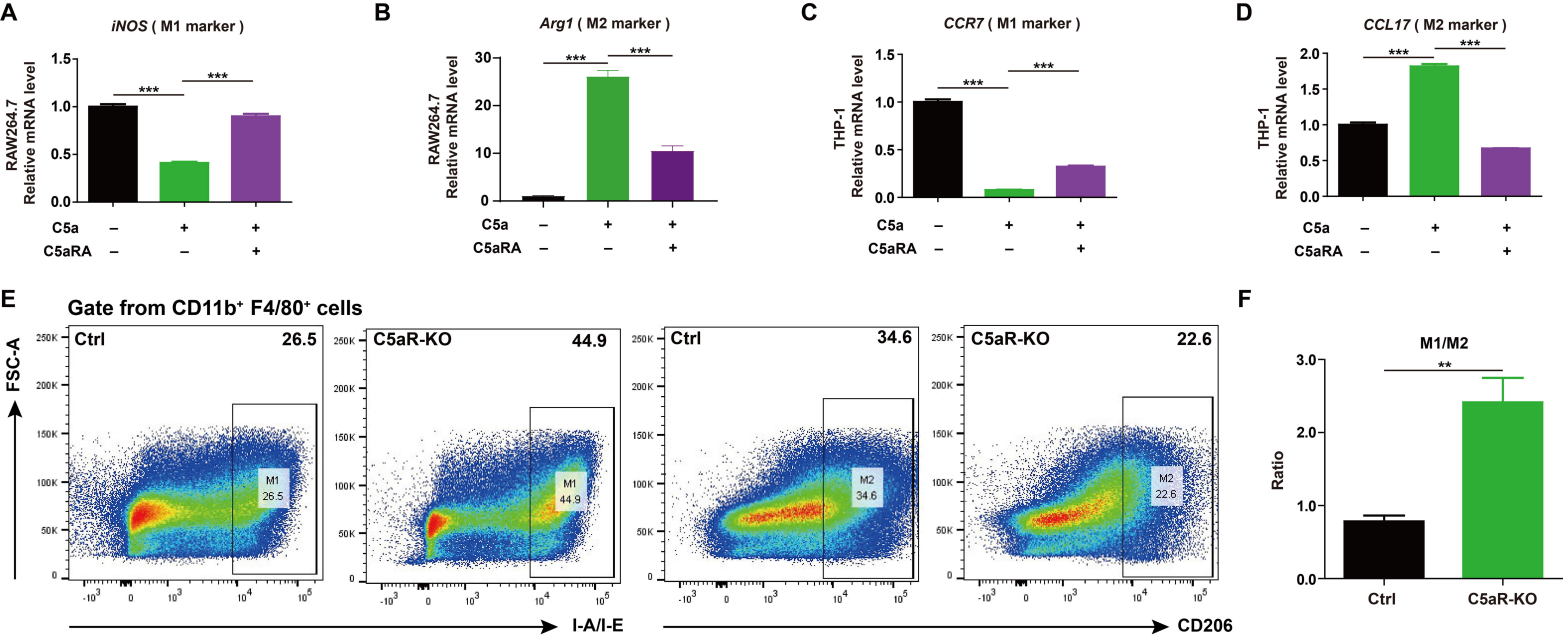
C5a-C5aR pathway promoted macrophage polarization to M2 phenotype. **(A-D)**, RAW264.7 and THP-1 cells were pre-incubated with 10 nM C5aRA for 60 min before exposure to 480 ng/mL C5a for 48h and then the expressions of M1 associated gene (iNOS, CCR7) and M2 associated gene (Arg1, CCL17) were measured by RT-qPCR. The expression level of mRNA was normalized to β-actin (n=3). **(E, F)**, Flow cytometry analysis of IA-IE and CD206 expression in tumor-associated macrophages, representative flow cytometry diagram **(E)** and the ratio of M1/M2 macrophages **(F)**. Statistical differences are assessed by student’s t-test. Data were mean ± s.e.m. *, p < 0.05; **, p < 0.01; ***, p < 0.001.

It was reported C5a/C5aR pathway could lead tumor-associated macrophages polarization toward immunosuppressive phenotype in human ovarian cancer (23). As macrophages play a key role in shaping the immune state within the tumor microenvironment and cancer progression (24), it makes sense to test whether C5a/C5aR pathway could accelerate breast cancer progression by influencing macrophage polarization. The ex vivo results showed C5a/C5aR pathway could promote macrophage M2-like polarization in RAW264.7 and THP-1 cells (**Figures 3A-D**). The flow cytometry analysis also demonstrated that C5aR deficiency decreased M2-like macrophages but increased that of M1-phenotype macrophages among tumor associated macrophages *in vivo* (**Figures 3E, F**).

Taken together, all these results demonstrated that C5a/C5aR pathway promoted breast cancer progression by leading cancer cell ferroptosis resistance and macrophage polarization towards M2 phenotype. Blocking this pathway could be a therapeutic strategy for breast cancer.

### 3.3 PEG-Fe_3_O_4_ displayed anti-tumor efficacy by ferroptosis and macrophage repolarization

As blocking C5a/C5aR pathway may inhibit breast cancer progression by reducing ferroptosis resistance. Combined C5a receptor antagonists (C5aRA) with ferroptosis inducer might be a novel therapy strategy for breast cancer.

For iron nanoparticles have been wildly used in leading cancer cell ferroptosis, and mesoporous hollow iron nanoparticles have great potential for it also can be used as drug carrier (6, 25), we decided to test whether mesoporous hollow iron nanoparticles could be combined with C5aRA for breast cancer treatment.

**Figure 4:**
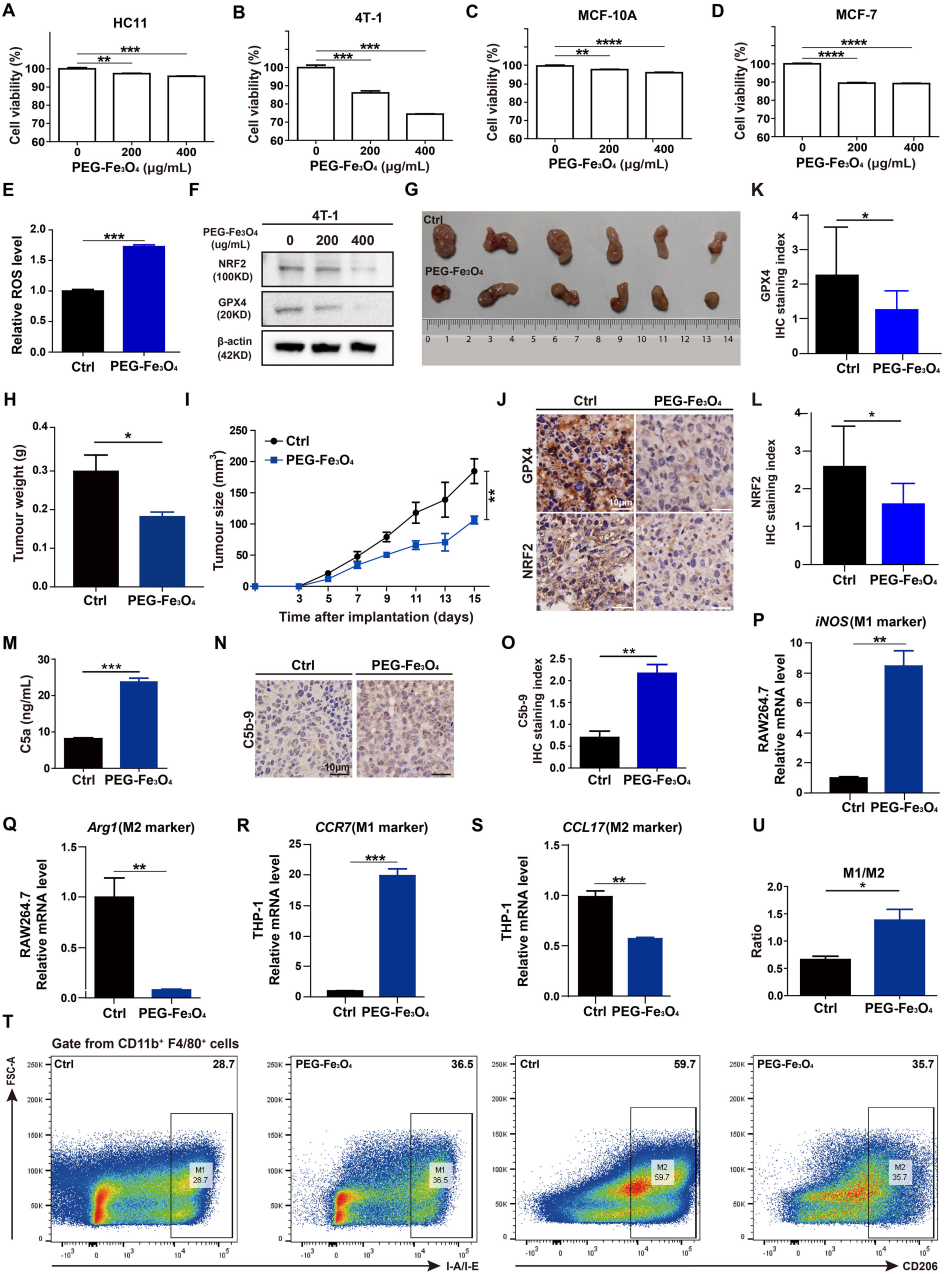
Evaluations of anti-tumor effect of mesoporous hollow PEG-Fe_3_O_4_. **(A-D)**, Cell viability of mouse breast cancer cell line 4T-1, non-malignant mouse fibroblast cell line HC11, human breast cancer cell line MCF-7, human normal mammary epithelial cell line MCF-10A were determined after treatment with PEG-Fe_3_O_4_ for 48h (n=4). Statistical differences are assessed by one-way ANOVA. Data were mean ± s.e.m. *, p < 0.05; **, p < 0.01; ***, p < 0.001; ****, p < 0.001. **(E)**, ROS level analysis of 4T-1 cells treated with PEG-Fe_3_O_4_ (200ug/mL) for 48h (n=3). **(F)**, Immunoblotting of NRF2 and GPX4 in 4T-1 cells after treatment with PEG-Fe_3_O_4_ for 48h. **(G-I)**, mice were implanted with 1.0 × 10^6^ 4T-1 cells in the mammary fat pad with and without PEG-Fe_3_O_4_ treatment. The tumor growth was measured by representative tumor images **(G)**, tumor weight **(H)**, and tumor size **(I)** (n=6). **(J-L)**, Immunohistochemistry and and statistical analyses of the GPX4 and NRF2 expression in tumor tissues from 4T-1 xenografts and 4T-1 xenografts with PEG-Fe_3_O_4_ treatment. **(M)**, Quantification of complement activation products C5a by ELISA: levels of anaphylatoxin C5a in mouse plasma (administered with PBS or PEG-Fe_3_O_4_) (n=10). **(N, O)**, Immunohistochemistry and statistical analyse of C5b-9 in tumor tissues from 4T-1 xenografts (left) and 4T-1 xenografts (right) with PEG-Fe_3_O_4_. **(P-S)**, Expressions of M1 associated gene (iNOS, CCR7) and M2 associated gene (Arg1, CCL17) were measured by RT-qPCR in RAW264.7 and THP-1 treated with PEG-Fe_3_O_4_ for 48h, and the expression levels of mRNA were normalized to β-actin (n=3). **(T, U)**, Flow cytometry analysis of IA-IE and CD206 expression in tumor-associated macrophages, representative flow cytometry diagram **(T)** and the ratio of M1/M2 macrophages **(U)**. Statistical differences are assessed by student’s t-test. Data were mean ± s.e.m. *, p < 0.05; **, p < 0.01; ***, p < 0.001.

First, to evaluate whether the cytotoxic effect of PEG-Fe_3_O_4_ was specific for cancer cells, mouse breast cancer cell line 4T-1, non-malignant mouse fibroblast cell line HC11, human breast cancer cell line MCF-7, human normal mammary epithelial cell line MCF-10A were treated with various doses of PEG-Fe_3_O_4_ for 48h and then subjected to MTT assay (**Figures 4A-D**). Although the cell viability was decreased by PEG-Fe_3_O_4_ treatment both in cancer cell lines and non-malignant cell lines, PEG-Fe_3_O_4_ was far more toxic to cancer cells than normal cells. When treated these cells with PEG-Fe_3_O_4_ at the concentration of 200ug/mL, the cell viability of 4T-1 decreased 26%, but only 4% for HC11; Consistent with this result, the cell viability of MCF-7 decreased 11% but only 4% for MCF-10A. To explore whether the inhibition of cell viability was through ferroptosis, the intracellular reactive oxygen species (ROS) level in cancer cells after PEG-Fe_3_O_4_ treatment was determined. PEG-Fe_3_O_4_ treatment drastically increased the intracellular ROS level in 4T-1 cells (**Figure 4E**). Besides, NRF2 and GPX4 were both reduced in 4T-1 cells after PEG-Fe_3_O_4_ treatment (**Figure 4F**). Together, these findings revealed that mesoporous hollow PEG-Fe_3_O_4_ showed cancer cell specific cytotoxicity, and it could induce ROS generation and cancer cell ferroptosis.

Then, the *in vivo* anti-tumor effect of mesoporous hollow PEG-Fe_3_O_4_ was tested in 4T-1 subcutaneous xenograft animal models. As showed in **Figures 4G-I**, tumor volume and tumor weight of 4T-1 xenografts were significantly reduced after intravenous (i.v.) injection of PEG-Fe_3_O_4_ as compared to the 0.9% NaCl solution control. The IHC staining of xenografts tumor tissues showed that the protein levels of NRF2 and GPX4 of xenograft tumor tissues were significantly reduced by PEG-Fe_3_O_4_ exposure (**Figures 4J-L**).

Besides ferroptosis inducing ability, previous study also showed that mesoporous hollow PEG-Fe_3_O_4_ could regulate macrophages polarization in neural system (7). We evaluated the ability of PEG-Fe_3_O_4_ to modulate macrophage polarization *in vitro*. PEG-Fe_3_O_4_ iron oxide nanoparticles were added to RAW264.7 cells for 48h and the RT-qPCR results revealed that PEG-Fe_3_O_4_ treatment promoted the M1-polarization induction-derived overexpression of *iNOS* (**Figure 4P**), while attenuated the expression of the M2 polarization gene *Arg1* (**Figure 4Q**). To further evaluate whether macrophages can be reprogrammed by PEG-Fe_3_O_4_, PMA pre-treated THP-1 macrophages were treated with PEG-Fe_3_O_4_, the RT-qPCR results also demonstrated that PEG-Fe_3_O_4_ was able to enhance the expression of M1 marker *CCR7* but reduced the level of M2 marker *CCL17* in THP-1 macrophages (**Figures 4R, S**). All these data suggested that PEG-Fe_3_O_4_ could modulate macrophage polarization towards M1 phenotype.

Next, to verify whether it could reprogram macrophages in breast cancer model, we collected the endpoint 4T-1 xenograft from the mice to analyze tumor-infiltrating macrophages by flow cytometry analysis (**Figure 4T**). The flow cytometry analysis showed that PEG-Fe_3_O_4_ treatment increased the ratio of M1-like macrophages/M2-like macrophages (**Figure 4U**).

These results demonstrated that PEG-Fe_3_O_4_ could inhibit breast cancer development through leading cancer cells ferroptosis and macrophage polarization to M1 phenotype. And for its drug carrier ability, loading it with C5aRA could be an effective breast cancer treatment strategy.

### 3.4 PEG-Fe_3_O_4_ could activate the complement system

Despite the anti-tumor effect of PEG-Fe_3_O_4_, it has been reported that iron nanoparticles could induce complement system activation, which may bring negative effect on tumor inhibition (14). To test whether the mesoporous hollow PEG-Fe_3_O_4_ could activate C5a/C5aR pathway, female BALB/C mice were administered with PEG-Fe_3_O_4_. After exposure, PEG-Fe_3_O_4_ induced 2.9-fold increment of C5a (**Figure 4M**). Under pathological conditions, after 4T-1 xenografts mice were treated with PEG-Fe_3_O_4_, the IHC staining of xenografts tumor tissues showed that the level of C5b-9 of xenograft tumor tissues was significantly increased (**Figures 4N, O**). All these results showed that PEG-Fe_3_O_4_ could induce complement system activation under both normal physiological and pathological conditions.

### 3.5 Synthesis and characterization of PEG-Fe_3_O_4_@C5aRA

**Figure 5:**
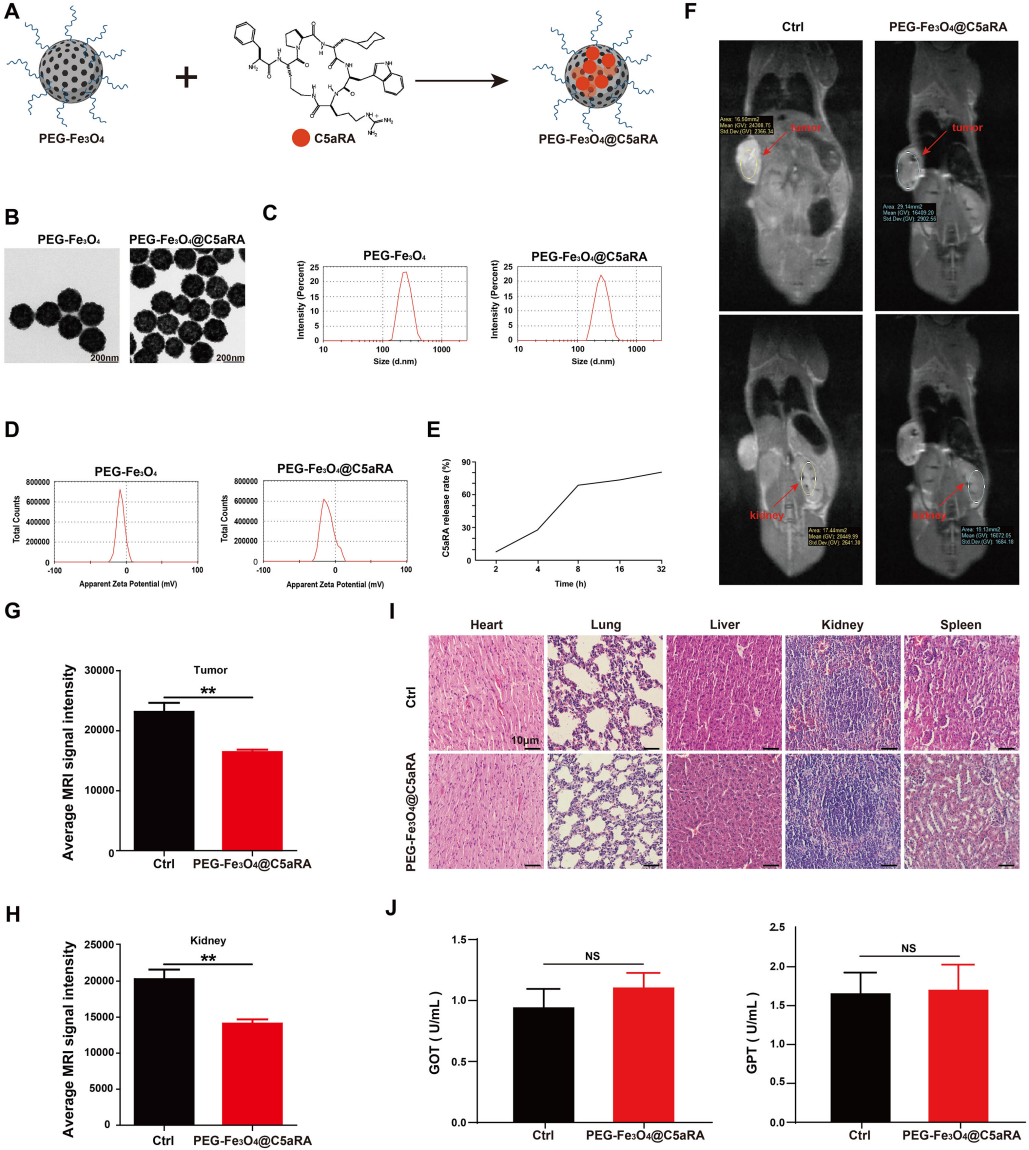
Construction and characterization of PEG-Fe_3_O_4_@C5aRA. **(A)**, Construction of PEG-Fe_3_O_4_@C5aRA. **(B-D)**, TEM images, size distribution and zeta potential of PEG-Fe_3_O_4_ and PEG-Fe_3_O_4_@C5aRA. **(E)**, C5aRA release curve under the microenvironment PH condition outside the tumor (PH=6.8). **(F)**, *In vivo* T2-weighted MR images 4 hours after administration. **(G, H)**, the relative signal intensity in tumor and kidney after treatment (n=4). **(I)**, H&E staining of main organs harvested from control and PEG-Fe_3_O_4_@C5aRA treatment groups. **(J)**, Main liver function index GOT and GPT of serum from control and PEG-Fe_3_O_4_@C5aRA treatment mice. Statistical differences are assessed by student’s t-test. Data were mean ± s.e.m. **, p < 0.01.

Based on the forward results, we postulated that anti-C5a/C5aR pathway treatment may enhance the anti-tumor ability of PEG-Fe_3_O_4_. As an efficient drug delivery carrier, it is possible to load mesoporous hollow PEG-Fe_3_O_4_ with C5aRA. As showed in **Figure 5A**, Fe_3_O_4_@C5aRA was constructed by loading mesoporous hollow PEG-Fe_3_O_4_ with C5aRA. The drug loading capacity (DLC) and encapsulation efficiency (EE) of PEG-Fe_3_O_4_@C5aRA were quantified by reading the absorbance of C5aRA at 360 nm. The DLC and EE were calculated to be 12.29% and 70.05%. Then, the *in vitro* release behavior of C5aRA from PEG-Fe_3_O_4_@C5aRA was determined at 37°C under the microenvironment PH condition outside the tumor (PH=6.8). According to the detection data (**Figure 5E**), the release of polypeptides was concentrated in the first 8 hours. The release amount reached nearly 70% in the first 8 hours and the packaged polypeptides were slowly released over time. As displayed in **Figure 5B**, loading PEG-Fe_3_O_4_ with C5aRA had no influence on the PEG-Fe_3_O_4_ iron oxide nanoparticles size. The transmission electron microscopy (TEM) images showed PEG-Fe_3_O_4_ and PEG-Fe_3_O_4_@C5aRA nanoparticles with a shape close to spherical and average diameter of 220nm. Dynamic light scattering (DLS) data indicated that PEG-Fe_3_O_4_@C5aRA had a larger hydrodynamic diameter (283.3 nm), compared to PEG-Fe_3_O_4_ (244.9 nm) (**Figure 5C**). The polydispersity index (PDI) values of PEG-Fe_3_O_4_ (0.207) and PEG-Fe_3_O_4_@C5aRA (0.245) were all < 0.3, indicated that the materials had good dispersibility. The average zeta potential of PEG-Fe_3_O_4_@C5aRA (−10.7 mV) was lower than that of bare PEG-Fe_3_O_4_ (−7.45 mV), reflecting the successful loading of C5aRA on PEG-Fe_3_O_4_ nanoparticles (**Figure 5D**).

### 3.6 *In vivo* imaging ability of PEG-Fe_3_O_4_@C5aRA and toxicity evaluation

To test whether PEG-Fe_3_O_4_@C5aRA could accumulate in tumor, 4T-1 xenograft mice were intravenous injection with PEG-Fe_3_O_4_@C5aRA, the control group were treated with 0.9% NaCl solution. As presented in **Figure 5F**, an obvious darkening effect in the tumor and kidney was observed in T_2_-weighted MR images. The darkening of the MR images in the mouse organs after injection was also confirmed quantitatively. The signal intensities of tumor and kidney were reduced remarkably after the administration of PEG-Fe_3_O_4_@C5aRA. It is no doubt PEG-Fe_3_O_4_@C5aRA could reach kidney, and the same change of signal intensities in tumor and kidney could demonstrate that PEG-Fe_3_O_4_@C5aRA accumulated in tumor 4 hours post injection (**Figures 5G, H**).

We also investigated the systematic toxicity of PEG-Fe_3_O_4_@C5aRA *in vivo* by H&E staining sections. As shown in **Figure 5I**, there was no observable pathological damage in the H&E staining sections in major organs of mice after different treatments. Furthermore, biochemical analysis of blood samples was conducted after different treatments for 48 h. The results revealed that the primary liver function markers: glutamic oxaloacetic transaminase (GOT) and glutamic pyruvic transaminase (GPT) (**Figure 5J**) remaining in a standard range. Those results revealed excellent biocompatibility and biosafety of PEG-Fe_3_O_4_@C5aRA.

### 3.7 PEG-Fe_3_O_4_@C5aRA displayed enhanced anti-tumor ability

**Figure 6:**
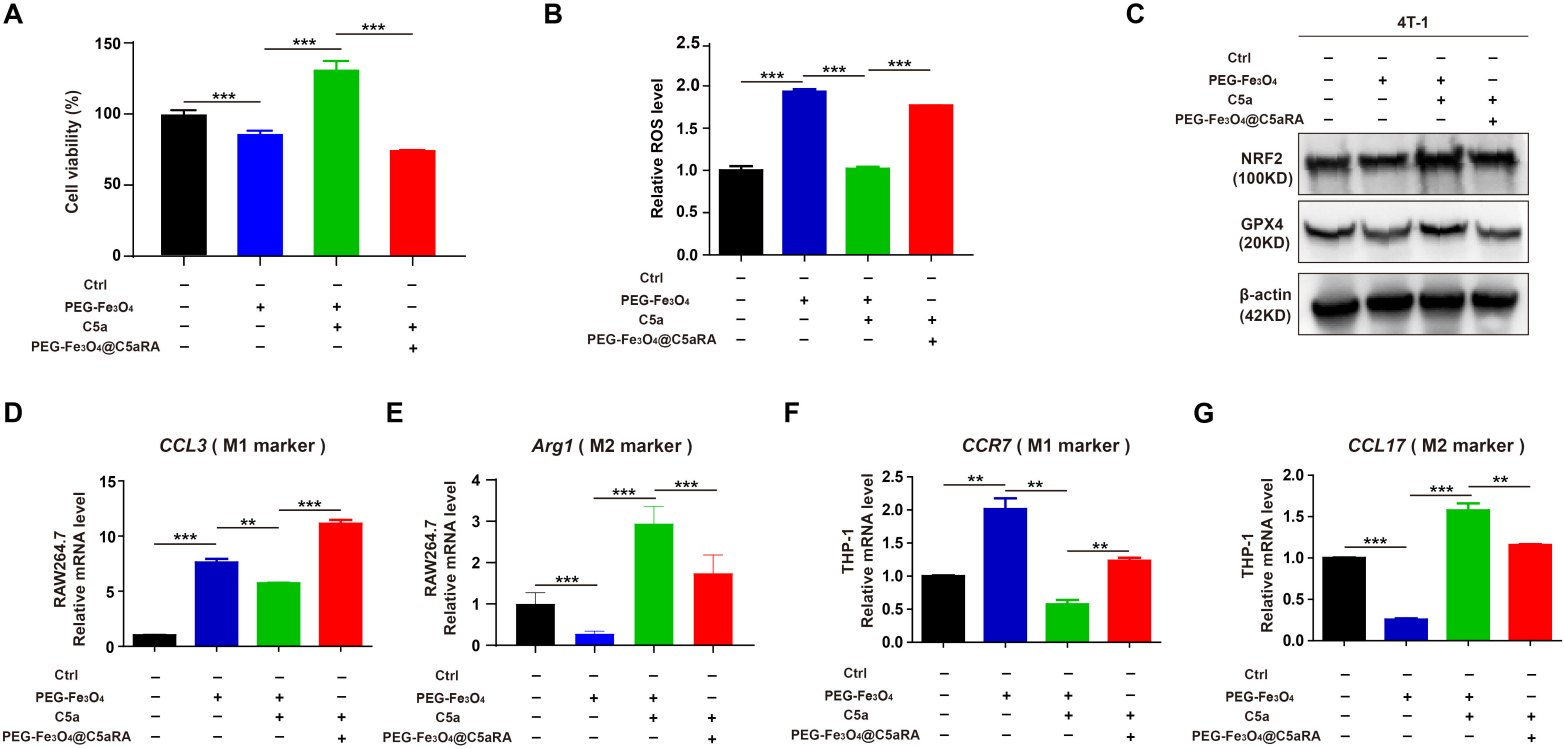
PEG-Fe_3_O_4_@C5aRA displayed enhanced ability by inducing cancer cell ferroptosis and macrophage polarization to M1 phenotype *in vitro*. **(A-C)**, Relative cell viability **(A)**, ROS level **(B)**, NRF2 and GPX4 expression **(C)** analysis of 4T-1 cells exposured to PEG-Fe_3_O_4_ or recombinant murine C5a plus PEG-Fe_3_O_4_ or C5a plus PEG-Fe_3_O_4_@C5aRA for 48h. D-G, Expressions of M1 associated gene (CCL3, CCR7) and M2 associated gene (Arg1, CCL17) were measured by RT-qPCR in RAW264.7 and THP-1 treated with PEG-Fe_3_O_4_ or C5a plus PEG-Fe_3_O_4_ or C5a plus PEG-Fe_3_O_4_@C5aRA. The expression levels of mRNA were normalized to β-actin (n=3). Statistical differences are assessed by student’s t-test. Data were mean ± s.e.m. **, p < 0.01; ***, p < 0.001.

To determine whether PEG-Fe_3_O_4_@C5aRA displayed enhanced anti-tumor effect, 4T-1 cells were treated with PEG-Fe_3_O_4_, PEG-Fe_3_O_4_ plus C5a, and PEG-Fe_3_O_4_@C5aRA plus C5a. The MTT result showed that PEG-Fe_3_O_4_@C5aRA displayed stronger tumor inhibition effect (**Figure 6A**). C5a decreased the generation of ROS induced by Fe_3_O_4_ treatment, and C5aRA loaded in the PEG-Fe_3_O_4_ could reverse it (**Figure 6B**). Besides, NRF2 and GPX4 were both reduced in 4T-1 cells after PEG-Fe_3_O_4_@C5aRA treatment (**Figure 6C**). Together, all these results revealed that PEG-Fe_3_O_4_@C5aRA which combined PEG-Fe_3_O_4_ and C5aRA could contribute to 4T-1 cell ferroptosis, increasing ROS generation and down-regulation of NRF2 and GPX4. Besides, treated RAW264.7 and THP-1 cells with PEG-Fe_3_O_4_@C5aRA could promote macrophages polarization to M1 phenotype but decreased that of M2-like macrophages (**Figures 6D-G**).

**Figure 7:**
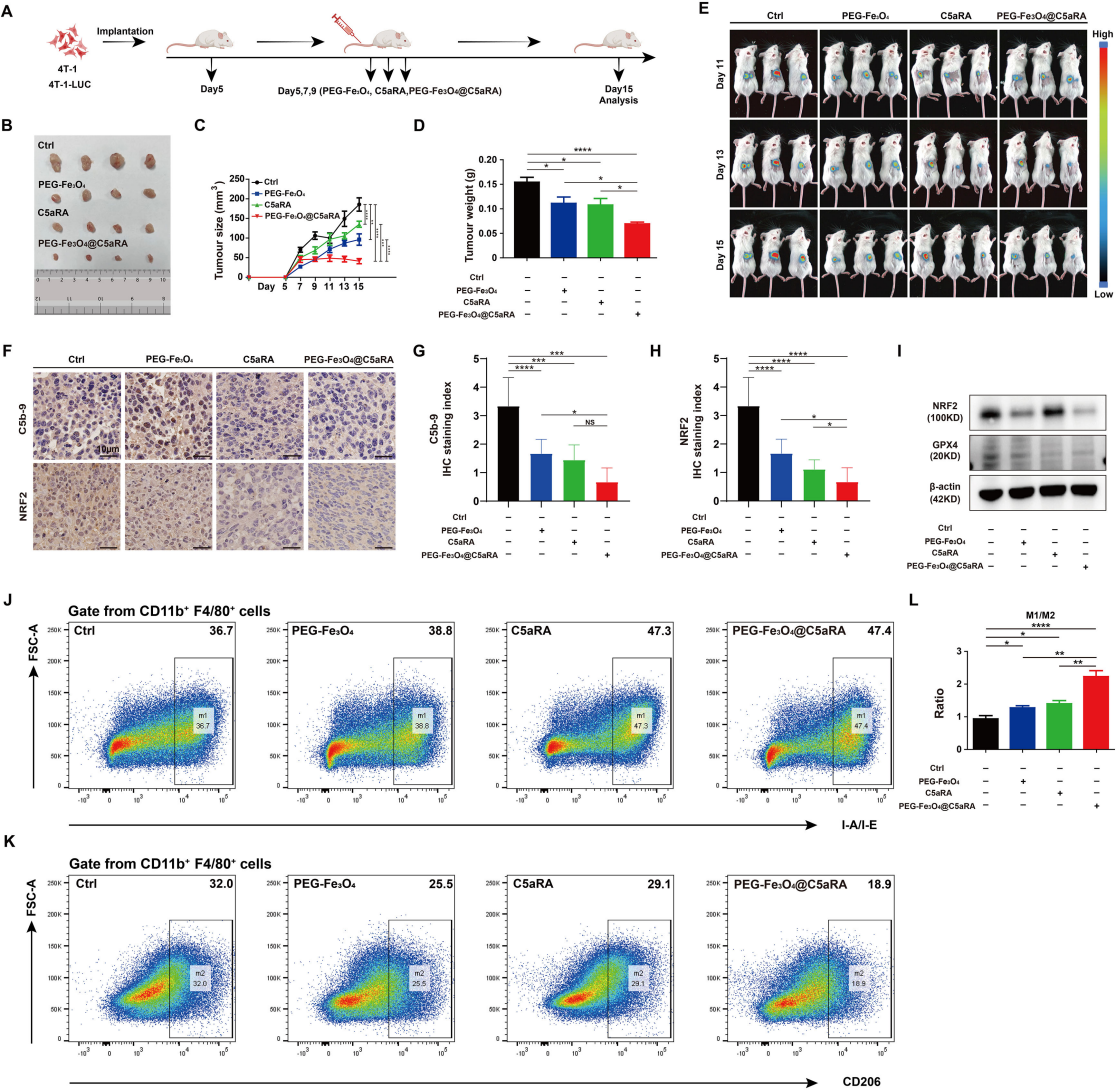
PEG-Fe_3_O_4_@C5aRA showed superior anti-tumor properties *in vivo*. BALB/C mice were inoculated with 1 × 10^6^ 4T-1 or 4T-1-LUC tumor cells, and then treated with PEG-Fe_3_O_4_, C5aRA or PEG-Fe_3_O_4_@C5aRA. PEG-Fe_3_O_4_ (20 mg/kg body weight, Day5, 7, 9), C5aRA (1 mg/kg body weight, Day5, 7, 9) or PEG-Fe_3_O4@C5aRA (20 mg/kg body weight, Day5, 7, 9) was injected intravenously. The detail of groups and treatments are shown in the figure **(A)**, the arrows indicated the timing of therapy. The tumor growth was measured by representative tumor images **(B)**, tumor size **(C)** and tumor weight **(D)** (n=4). **(E)**, Representative bioluminescence imaging during tumor progression (n=3). **(F-H)**, Immunohistochemistry staining and statistical analyses of C5b-9 and NRF2 in tumor tissues. **(I)**, Immunoblotting staining of the expression of NRF2 and GPX4 in tumor tissues. **(J-L)**, Flow cytometry analysis of IA-IE and CD206 expression in tumor-associated macrophages, representative flow cytometry diagram **(J, K)** and the ratio of M1/M2 macrophage **(L)**. Statistical differences are assessed by one-way ANOVA. Data were mean ± s.e.m. *, p < 0.05; **, p < 0.01; ***, p < 0.001, ****, p < 0.001.

To better evaluate the potential of combined therapy in a model that more closely resembles the clinical setting, we treated established 4T-1 tumors during a defined period (**Figure 7A**). In the subcutaneous 4T-1 model, treatment with PEG-Fe_3_O_4_@C5aRA resulted in a significant reduction of tumor growth, as compared with PEG-Fe_3_O_4_ or C5aRA treatment alone. By day 15, tumor volumes in mice treated with the PEG-Fe_3_O_4_@C5aRA were smaller than those in control group, mice treated with PEG-Fe_3_O_4_ or C5aRA groups (**Figures 7B-D**). The super anti-tumor effect of PEG-Fe_3_O_4_@C5aRA was further verified by *in vivo* monitor of the tumor growth using bioluminescence imaging. 4T-1-luciferase (4T-1-LUC) cells were inoculated into the back of BALB/C mice. Tumor growth at various stages was recorded by IVIS Spectrum *in vivo* optical imaging system (PerkinElmer) on day 11, 13 and 15 after 4T-1-LUC cells inoculation. The results have shown that the bioluminescence signals decreased significantly in the PEG-Fe_3_O_4_@C5aRA treatment group compared to the control and treatment alone groups (**Figure 7E**).

The western blot and IHC staining of xenografts tumor tissues showed that the protein levels of NRF2 and GPX4 of xenograft tumor tissues were significantly reduced by PEG-Fe_3_O_4_@C5aRA exposure. The C5b-9 stain result also proved that PEG-Fe_3_O_4_ activated complement system, loading it with C5aRA inhibited the activation. (**Figures 7F-I**). And the PEG-Fe_3_O_4_@C5aRA treatment resulted in a significant elevation of the ratio of M1 macrophage/M2 macrophage in TME (**Figures 7J-L**). From all these experiments, we concluded that that the PEG-Fe_3_O_4_@C5aRA exhibited superior antitumor efficacy.

## 4 Discussion

Although there are many effective therapies available for tumors, they also pose a significant threat to human health due to drug resistance and low patient response. Consequently, various cancer therapies have been developed, with a focus on inducing cancer cell death through different forms of cell death such as apoptosis, necrosis, necroptosis, and pyroptosis or aiming at reprogramming tumor microenvironment to anti-tumor state (10, 26). Recently, metal metabolism associated cell death like ferroptosis and cuproptosis have attracted increasing attentions in cancer therapy research area (27, 28). Ferroptosis is a reported mode of programmed cell death caused by the accumulation of iron-dependent lipid peroxidation (LPO) in cells (29). Since being found, ferroptosis has attracted considerable attention due to its potential role as a target for novel therapeutic anticancer strategies (30). Iron nanoparticles, such as ferumoxytol, Fe_3_O_4_ and Zero-valent-iron nanoparticles, have shown promising applications in cancer therapy by inducing cancer cell ferroptosis (31, 32).

In addition to targeting cancer cells, the tumor microenvironment also plays a significant role in tumor progression and is increasingly recognized as a promising cancer therapeutic target. This treatment focuses on reprogramming the immune microenvironment to identify and fight against tumor. Tumor-associated macrophages have aroused great interest in recent years as a therapeutic target because they are tumor-enriched immunosuppressor cells that influence tumor progression and metastasis (33). Promoting TAMs towards a pro-immunogenic profile has been proposed as an attractive therapeutic tool to enhance local anti-tumor immune responses. Research has demonstrated that iron nanoparticles can enhance macrophage-modulating cancer immunotherapies by promoting TAM polarization to M1 phenotype (34).

Although many traditional treatments for breast cancer are effective in destroying tumor cells, they always also harm normal cells, leading to negative effect that impact which will decrease patient’s quality of life (35). Besides, chemotherapies and radiotherapies are also unable to eliminate the critical cancer stem cells, which are protected by specific resistance mechanisms, resulting in the development of new tumors and metastases (36). Consequently, new strategies and drugs are urgently needed for breast cancer therapy. Nanoparticles, with the physic-chemical characteristics, like reduced toxicity, fine size, chemical composition and large surface-to-volume ratio, have been widely researched and served as a novel method for tumor diagnosis and treatment. Nonmetallic structured nanoparticles like Propolis nanoparticles developed by Ebru Nur Ay displayed cytotoxic and apoptotic effects on breast cancer cells (37). And metal nanoparticles (MNPs) based on metals like gold, silver, copper, iron, zinc have gained interests because their special characteristics for cancer therapy. Ferdane Danışman-Kalındemirtaş constructed 5FU-AgNPs, which showed antiproliferative effects on different breast cancer cells (38). Ceylan Hepokur also reported Capecitabine bonded silver particles significantly increased the number of early and late apoptotic cells on MCF-7 cells (39). Studies have also demonstrated that treatment with iron nanoparticles inhibited the growth of breast cancer cells (10, 40). In Ferdane Danışman-KalındemirtaşIn’s study, Albumin-Bound Fe (III)-S-Methyl-Thiosemi carbazones were successfully synthesized, and their anticancer effects were examined. These NPs enhanced the anticancer activity of Fe(III)-S-Methyl-Thiosemicarbazones on breast cancer cells (41). Our results are consistent with this finding that iron nanoparticles can effectively treat breast cancer with low side reactivity and high delivery. Although there are differences in the choice of delivery vectors, both showed good therapeutic effects on breast cancer. Additionally, an iron oxyhydroxide-based nano-system has been shown to inhibit the growth of breast cancer stem cells (9). All these studies suggested that iron nanoparticles-based therapy strategies may be a new direction for breast cancer therapy in the future.

However, iron nanoparticles alone are not sufficient to induce cancer cells lethal ferroptosis due to the presence of ferroptosis resistance. To survival, cancer cells developed a series of strategy to defeat ferroptosis. Like in head and neck squamous cell carcinoma, Interleukin-6, a representative inflammatory cytokine, could induce ferroptosis resistance by IL-6/STAT3/xCT axis (42). So, further analysis of the mechanism of ferroptosis resistance is important for the development of therapies based on iron particles induced ferroptosis.

Accumulating evidence suggests that the complement system plays a key role in regulating cancer immunity, thus involving, to varying degree, in tumor initiation and development (16, 43). And C5a/C5aR pathway has been associated with tumor progression and poor prognosis in breast cancer patients (17, 18). Tumor-promoting effects of C5a have also been reported in other murine cancer models, including breast, cervical, lung, ovarian, colorectal and melanoma (44–47). Previous studies have explored the role of the C5a/C5aR pathway in breast cancer progression. Maciej M. Markiewski demonstrated that ribosomal protein S19 interacts with C5aR1 and promotes breast cancer growth by facilitating the recruitment of MDSCs to tumors (48). Moreover, Baochi Ou showed that C5aR1^+^ neutrophils enhance the glycolytic capacity of breast cancer cells through the ERK1/2-WTAP-ENO1 signaling pathway (49). Xi Li provided evidence that C5aR1 inhibition reprograms tumor-associated macrophages in two murine breast cancer models: one intrinsically sensitive to PARP inhibitors (T22) and the other resistant (T127). In these models, Rps19/C5aR1 signaling is selectively elevated in TAM_C3, which predominantly expresses genes associated with an anti-inflammatory/protumor state (50).However, whether C5a/C5aR pathway are involved in ferroptosis during breast cancer carcinogenesis remains unclear. Previous studies have shown that C3a and C5a increased the expression of NRF2 in multiple myeloma cell line U266 (19). As NRF2 expression could inhibit ferroptosis, we speculated that this pathway could participate in ferroptosis resistance. Our study showed that activation of C5a/C5aR pathway resulted in ferroptosis resistance and increased TAMs polarization to M1 phenotype.

Based on these observations, we proposed a novel combination strategy in which mesoporous hollow iron nanoparticles PEG-Fe_3_O_4_ was loaded with a drug aimed at inhibiting C5a/C5aR pathway. We demonstrated that PEG-Fe_3_O_4_ synergizes with C5aRA in 4T-1 xenograft breast cancer model resulted in enhanced cell ferroptosis and TAMs polarization to M1 phenotype. Additionally, C5aRA blocked the negative effect of complement activation caused by PEG-Fe_3_O_4_ treatment.

The rationality and most plausible point of PEG-Fe_3_O_4_@C5aRA is that the combination of PEG-Fe_3_O_4_ and C5aRA could not only eliminate the negative therapeutic effects of complement activation induced by PEG-Fe_3_O_4_ treatment, but also enhance PEG-Fe_3_O_4_ caused cancer cell ferroptosis and TAMs polarization to M1 phenotype.

It has been confirmed that the size of iron nanoparticles plays a significant role when they are used in biomedicine. To avoid rapid filtration by the spleen and liver and to prolong the circulation time in the blood, the size of these nanoparticles should be small, preferably below 200nm (51). Like in Ferdane Danışman-Kalındemirtaş’s study, albumin-bound Fe(III)-S-Methylthiosemicarbazones was developed for breast cancer thepapy. And the average particle size was roughly 84.62 nm (41). However, the size of the PEG-Fe_3_O_4_@C5aRA nanoparticles is 220nm, which raised doubts about their ability to accumulate in tumor. Since PEG-Fe_3_O_4_ nanoparticles possess MRI capability, we demonstrated PEG-Fe_3_O_4_@C5aRA can reach tumor site and bring anti-tumor effect. Consistent with our findings, Qin Jiang et al. reported an iron nanoparticles Fe_3_O_4_-SAS@PLT with the size of 268.9 ± 8.9 nm, it mediated ferroptosis enhancing cancer immunotherapy in a metastasis model by injecting 4T1-luc cells into the tail vein of BALB/C mice intravenously, and observed the accumulation of Fe_3_O_4_-SAS@PLT in metastatic tumor with *in vivo* imaging (52). Mengqi Zhang et al. constructed a drug delivery system DOX@HFON by loading a hollow mesoporous ferric oxide nanoparticle with DOX, this system displayed anti-tumor effect in MDA-MB-231 xenografts breast cancer model in nude mice, and the MRI result also showed it could accumulate in the tumor although its size is 486 ± 63 nm (53). Although PEG-Fe_3_O_4_@C5aRA can accumulate in tumor, the size means it could filtrate by the spleen, kidney and liver. So, methods like introducing an exogenous magnetic field may enhance PEG-Fe_3_O_4_@C5aRA ability to accumulate in tumor and kill tumor cells.

In summary, we found C5a/C5aR pathway could promote breast cancer progression by inducing ferroptosis resistance and macrophage polarization to M2 phenotype. Based on this, a novel anti-tumor system PEG-Fe_3_O_4_@C5aRA was designed by loading PEG-Fe_3_O_4_ with C5aRA. PEG-Fe_3_O_4_@C5aRA showed a significant therapeutic efficacy on tumor-bearing mice. Our study provided an innovative approach to amplify iron nanoparticles anti-tumor effect by inhibiting complement activation to enhance ferroptosis and macrophage polarization to M1 phenotype, opening the door to designing superior iron nanoparticles-based therapy for tumor treatment.

## Data Availability

The original contributions presented in the study are included in the article/supplementary materials, further inquiries can be directed to the corresponding authors.
